# Barriers and facilitators of messaging platforms as a means of maternal support and care in rural communities: A systematic review

**DOI:** 10.1371/journal.pone.0336168

**Published:** 2025-12-05

**Authors:** Shahreen Rahman, Asua Okolie, Dianne Bryant, Edward Kwabena Ameyaw, Obidimma Ezezika

**Affiliations:** 1 Global Health & Innovation Lab, School of Health Studies, Faculty of Health Sciences, Western University, London, Ontario, Canada; 2 School of Physical Therapy, Faculty of Health Sciences, Western University, London, Ontario, Canada; 3 Institute of Policy Studies and School of Graduate Studies, Lingnan University, Tuen Mun, Hong KongChina; University of Porto Faculty of Medicine: Universidade do Porto Faculdade de Medicina, PORTUGAL

## Abstract

Improving maternal support in rural communities through messaging platforms can be a crucial strategy for enhancing antenatal visit rates and improving maternal health outcomes. Currently, there is a significant gap in maternal healthcare access in rural areas, where pregnant women often face barriers such as distance to healthcare facilities, lack of healthcare providers, and limited access to educational resources. This review explored the barriers and facilitators to providing maternal support through messaging platforms. A protocol for this systematic review was developed and registered in the PROSPERO international prospective register of systematic reviews database (Registration number: CRD42023492705). A comprehensive search was conducted on OVID MEDLINE, OVID EMBASE, EBSCO CINAHL, and SCOPUS, focusing on primary research articles published between January 1, 2003, and January 30, 2025. Eligibility criteria required studies to report on maternal support utilizing messaging platforms in rural populations globally. The search strategy yielded a total of 665 studies, of which 15 studies met the eligibility criteria. The quality of eligible studies was evaluated using the Mixed Methods Appraisal Tool, and data were extracted from the articles and analyzed using the Consolidated Framework for Implementation Research. Evidence from the studies indicated that messaging interventions positively impacted maternal health knowledge, access to perinatal services, and communication. Significant barriers were identified, including network connectivity issues in rural areas, affordability of airtime and phones, storage capacity of phones, resistance based on religious beliefs, reliance on traditional birth attendants, and literacy challenges. Facilitators in the messaging interventions included the educational nature of the messages, tailored advice related to pregnancy stages, timely support from community health workers, and improved access to transport for healthcare visits. Tailoring interventions to specific community needs and incorporating educational elements can enhance the engagement and success of messaging platforms targeting maternal health. The identified barriers and facilitators can inform the effective use of messaging platforms by ensuring they are accessible, affordable, and culturally appropriate to support maternal health in rural communities.

**Trial registration:**

PROSPERO international prospective register of systematic reviews database (Registration number: CRD42023492705).

## Introduction

Maternal health is a critical area of focus in public health. The Sustainable Development Goals (SDGs), which are targets to improve social, economic and environmental outcomes globally, emphasize working towards good health. Specifically, SDG 3 focuses on maternal health with indicators 3.1.1 and 3.1.2, which seek to reduce the maternal mortality ratio (per 100,000 live births) and increase the proportion of births attended by skilled health personnel, respectively [1]. Antenatal care (ANC), along with skilled birth attendance (SBA) and postnatal care (PNC), are essential to improving the maternal mortality ratio and maternal health outcomes globally [2].

Adequate ANC is vital for maternal and fetal health, involving health monitoring, medical and social support, and health promotion [3]. ANC assists in identifying and managing potential health issues early, providing expectant mothers with the necessary information and support to maintain their health while reducing both preterm deliveries and adverse outcomes for preterm infants [4]. ANC has been proven to improve pregnancy and birth outcomes by helping to connect the mother to healthcare professionals and preventing anemia, obstructed labour and other obstetric emergencies [3].

While maternal health outcomes have improved globally, substantial disparities remain between both high-income countries (HICs) and low- and middle-income countries (LMICs), and between urban and rural communities [5]. In resource-limited settings that are prevalent in LMICs and rural communities globally, access to ANC and SBA is low, and barriers to accessibility and affordability of health care services are linked to limited health infrastructure, funding, technological issues, and disparities in health literacy [5,6]. The greatest inequities in access to ANC and SBA are reported in LMICs throughout Africa and Asia [5].

Rural communities are often disadvantaged due to geographical isolation and limited access to healthcare essentials for positive maternal health outcomes, such as regular checkups, access to skilled birth attendants, and emergency obstetric services [7]. In addition, rural areas, especially those in LMICs, often struggle with longer distances to travel for care and lower socio-economic conditions that further complicate access to necessary maternal healthcare [7]. These disparities can result in poorer maternal health outcomes in rural areas compared to their urban counterparts [8].

Messaging services, including SMS (short messaging service), EMS (enhanced messaging service), and MMS (multimedia messaging service), offer a promising solution for improving maternal health support in rural communities [9]. Applications that use these services can facilitate communication, disseminate vital health information, and provide guidance to pregnant women who might otherwise have limited access to healthcare resources [10]. For this review, “messaging platform” refers to any application that utilizes SMS, EMS, or MMS to send text and multimedia messages, providing a means to enhance maternal support by improving access to healthcare information, reminders for ANC visits, and educational content related to nutrition and early warning signs of complications [9].

Research has demonstrated the potential of mHealth interventions, including messaging platforms, to improve maternity health outcomes. Systematic reviews have examined the use of SMS in increasing ANC visits and SBA, highlighting the positive impact of these technologies in various settings as well as outlining how text messaging has been used to promote maternal and infant health [10,11]. Additionally, studies have explored the role of mobile technology in mHealth for antenatal care and mHealth interventions addressing maternal health and perinatal care [12–14]. However, a study has found that mHealth interventions for maternal health are skewed towards high-income countries, which may indicate that further research is needed in LMICs [15]. While these reviews indicate the benefits of mHealth, there is a gap in the literature specifically focusing on the use of messaging platforms for maternal support in rural communities.

This systematic review aims to fill that gap by investigating whether messaging platforms can successfully enhance maternal support in rural areas. The study will assess acceptance and usage rates of these platforms, maternal health outcomes when these platforms are made available, and identify barriers and facilitators to their implementation. By synthesizing findings from existing studies, this review seeks to provide comprehensive insights into the potential of messaging platforms to improve maternal health in rural settings, contributing to better health outcomes for mothers and infants in underserved regions.

## Methods

**Fig 1:**
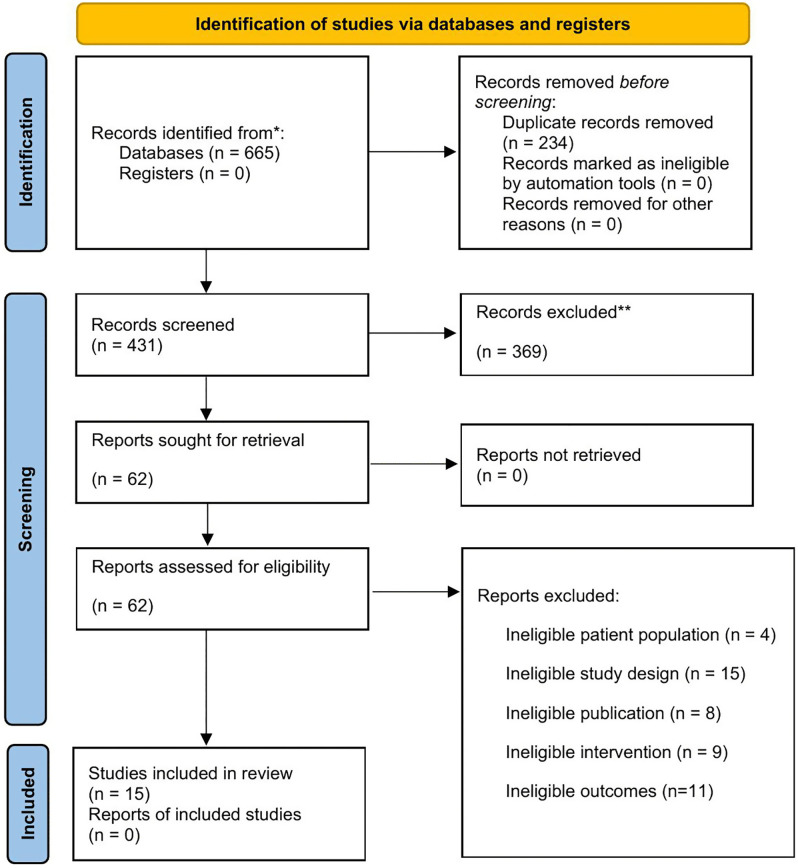
PRISMA-Flowchart.

The Preferred Reporting Items for Systematic Reviews and Meta-Analyses (PRISMA) 2020 was used in presenting this systematic review, which can be found in S1 Table PRISMA Checklist [16]. A protocol for this study is registered in the International Prospective Register of Systematic Reviews, PROSPERO CRD42023492705. Fig 1 shows the screening process using the PRISMA flowchart.

### Search strategy

A comprehensive search was conducted of OVID MEDLINE, OVID EMBASE, EBSCO CINAHL, and SCOPUS according to a search strategy created with assistance from an academic health sciences librarian (MS). The search was executed on January 30^th^, 2025. The results were excluded if the publication date was restricted between January 1^st^, 2003, to January 30^th^, 2025, since the majority of mHealth interventions were within the last 2 decades. The search strings used in each database are provided in S1 Appendix Search Strings.

### Eligibility criteria

To be eligible for the review, the studies needed to be primary research articles published between January 1^st^, 2003, and January 30^th^, 2025. Studies needed to report on maternal support utilizing messaging platforms as the intervention, addressing a rural population anywhere globally, with participants of any age. Studies were included with no limitations on language. The eligibility criteria are presented in Table 1.

**Table 1:** Selection, inclusion and exclusion criteria for the preliminary analyses of title and abstract screening and full-text review.

Selection Criteria	Inclusion Criteria	Exclusion Criteria
*Publication Characteristics*		
Publication Type	Peer-reviewed journal articles	Non-original and peer reviewed articles, grey literature in any format other than dissertations or working papers and commentaries
Study Type	Primary (Research)	Secondary (Reviews)
Publication Date	2003-2025	Articles published before 2003
*Study Characteristics*		
Issue	Maternal Health and Support	Public Health, Population Health, Issues unrelated to Maternal Health and Support
Population	Rural Populations	Urban Populations
Intervention	Messaging Platforms	Maternal Health and Support interventions that do not use messaging platforms
Outcome	Discussion of barriers and facilitators, impact of the intervention and reports on improvement of maternal health outcomes	No discussion of barriers and facilitators, impact of the intervention and reports on improvement of maternal health outcomes

### Study selection

Screening for this review was completed using Covidence, a systematic review management tool, through a three-step process. First, pilot testing of the eligibility criteria was completed to ensure consensus between the team members, after which all article titles and abstracts were screened independently and in duplicate by two reviewers (SR and AO). All studies captured by the search had English titles and abstracts. Conflicts were resolved through discussion between the reviewers. In the next step, the full texts of each study were located and imported into Covidence, following which the two reviewers completed full-text screening independently. Conflicts were resolved through group discussions.

### Data extraction

Following a pilot test of the data extraction templates, data extraction for the included articles was conducted independently by two reviewers. The templates captured the following items: authors, year of publication, study title, country, study design, population, study objectives, evidence of impact, barriers and facilitators, the respective excerpts, and Consolidated Framework for Implementation Research (CFIR) constructs. Conflicts in data extraction were resolved through group discussion. Conflicts in identifying evidence of impact included cases where one reviewer found a different instance where impact was demonstrated than the other reviewer. Conflicts for barriers and facilitators included cases where a reviewer either identified different barriers or facilitators from the other reviewer or labelled the barrier or facilitator differently.

A study characteristics table was created to compile all descriptive information. Data related to the barriers and facilitators were compiled into two consensus documents that consisted of the name of the barrier or facilitator that was extracted from each study, an excerpt that demonstrated the barrier or facilitator, along with the country and author for the study and the CFIR construct and domain that aligned with the barrier or facilitator. These documents were used to create collapsed tables for the barriers and facilitators, respectively. One team member grouped barriers and facilitators with the corresponding studies that had identified them, including excerpts that demonstrated the barrier or facilitator in the study.

### Data synthesis

In this review, “impact” was defined as any significant change in maternal health outcomes resulting from the use of messaging platforms. This included both measurable health outcomes (e.g., increased ANC visits, improved knowledge) and qualitative outcomes (e.g., satisfaction or perceived benefits). When studies presented mixed findings, all reported impacts were included in the analysis. For qualitative studies, impact was assessed based on participants’ narratives or themes indicating changes in attitudes, behaviours, or experiences linked to the intervention.

The CFIR framework was selected for its comprehensive structure, enabling the analysis of individual, organizational, and system-level factors influencing implementation. Unlike frameworks that focus more narrowly on behaviour change or outcome evaluation, CFIR is better suited for capturing the contextual barriers and facilitators present for maternal mHealth messaging interventions. Two team members used the CFIR framework to categorize each barrier and facilitator identified through data extraction. A pilot test was conducted with the first three barriers and facilitators, followed by a consensus meeting to review the work, during which any inconsistencies were clarified to ensure they could be mitigated. After this, the remaining barriers and facilitators were organized using the framework, with any conflicts being resolved through group discussion. The final consensus meeting was utilized to produce a table of the results with excerpts from the studies. A thematic analysis, which is the process of identifying, organizing and interpreting themes, was then conducted by two reviewers on the barriers and facilitators [17,18]. The themes emerged through an inductive process guided by a phenomenological lens, focusing on lived experiences with messaging interventions from pregnant women, healthcare workers and implementers. The two reviewers were deeply immersed in the data, reviewing quotes and reported outcomes, conducting open coding of extracted findings, and refining themes through discussions. By engaging with the findings across studies through this process, recurring patterns and salient concepts were identified. Outcome reporting (positive, negative, or neutral) was noted to support a balanced interpretation of the intervention’s effects across included studies.

### Quality assessment

The Mixed-Methods Appraisal Tool (MMAT) was used by two reviewers independently to assess the quality of the eligible studies [19]. Discrepancies between the reviewers were resolved through group discussions. The MMAT was selected because it allows for the appraisal of all three study types within a single tool, ensuring consistency and comparability across all studies. It evaluates studies based on clear criteria, including the coherence between research questions, data collection methods, sources of evidence, and interpretations. The MMAT assessment is included in S2 Table MMAT.

## Results

Study characteristics, including study design, year of publication, countries, participants, objectives and evidence of impact, were analyzed from eligible studies in this systematic review.

### Types of Study/Methods

The studies included in this systematic review consisted of qualitative (n = 3) [20–22], quantitative (n = 4) [23–26], and mixed methods (n = 8) [27–34]. Details are shown in S3 Table Study Characteristics.

In the qualitative studies (n = 3), data collection methods included in-depth interviews (n = 2) [21,22], document reviews (n = 1) [20], field observations (n = 1) [20], and focus group discussions (n = 2) [20,21]. In the quantitative studies (n = 4), methods included randomized controlled trials (n = 2) [25,26], a randomized parallel, two-arm standard of care-controlled feasibility trial (n = 1) [23], and a non-randomized intervention study with self-reported demographic information (n = 1) [24].

### Language

The majority of the articles captured by the search were in English (n = 429), with the search across all databases capturing only two studies in other languages that were specifically in French (n = 2). The two French studies were screened and were found not to meet the eligibility criteria during the title and abstract screening [35,36].

### Year of publication

The included articles were published as early as 2014 and as recently as 2023. The bulk of the articles used in our study were published between 2018 and 2020 (n = 8), with the most articles being published in 2018 (n = 5) [20,24,25,27,29]. The fewest articles were published in 2014 (n = 1) [28] and 2020 (n = 1) [34].

### Participants

The most frequently engaged target population across all studies in this review was pregnant women (n = 9) [20,22,24–27,29,33,34]. Other participants included those in maternal and child health contexts. For instance, mothers, including lactating mothers and postpartum women who had received the intervention, were recruited across several studies (n = 5) [20,22,29,31,33]. Health workers, community leaders, and community health workers, who are healthcare providers that receive lower levels of education than certified healthcare providers and live in the community they serve, were also significant participants (n = 3) [21,30,33]. Household members, both male and female, and caregivers were included in some studies (n = 2) [28,32]. Additionally, married women, regardless of their pregnancy status, were included (n = 1) [30]. Specific demographic groups, such as rural pregnant women who were positive for depression (n = 1) and women initiating breastfeeding within 24 hours of giving birth on antiretroviral treatment (n = 1), were also targeted [23,29]. All participants were from rural areas, ensuring a focused examination of rural maternal health dynamics.

### Countries

**Fig 2:**
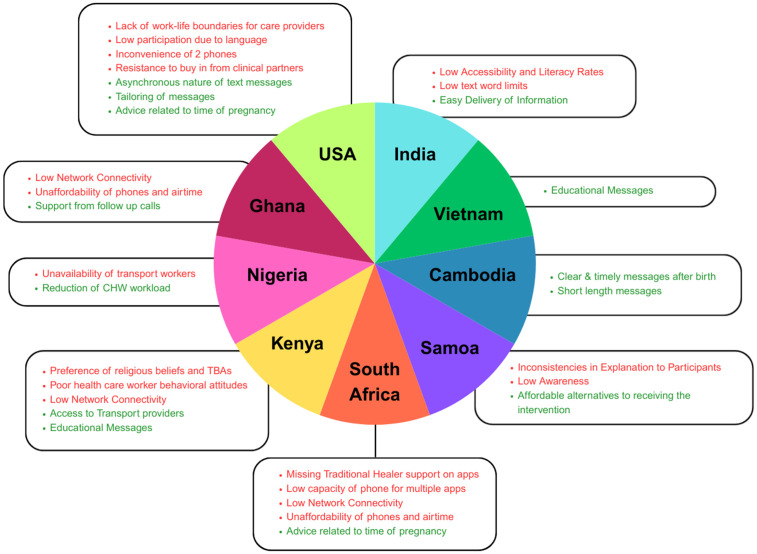
Barriers & Facilitators to mHealth Messaging Platforms in Rural Populations by Country.

A total of 10 countries were included across all studies, including the United States [27,29], India [25,28], South Africa [21,23], Kenya [22,26,32], Nigeria [37], Vietnam [20], Cambodia [31], Uganda [24], Ghana [33], and Samoa [34]. The most frequently cited settings were Africa and Asia. The most frequently studied countries were the United States (n = 2), India (n = 2), South Africa (n = 2), and Kenya (n = 3) [21–23,25–29,32]. Fig 2 shows the barriers and facilitators emerging from each country.

The barrier and facilitators labels have been shortened for the purposes of the figure. (A) The barriers corresponding with the country are noted in red. (B) The facilitators corresponding with the country are noted in green.

### MMAT

The MMAT was used to organize and assess methodological quality according to the tool’s established criteria. S2 Table MMAT provides the results for all 15 studies. No articles were removed after the MMAT assessment.

### Evidence of impact

The evidence from the 15 studies demonstrates the supportive nature of messaging interventions in improving maternal and child health outcomes. Participants in numerous studies expressed high satisfaction with the interventions, reporting that they found the messages helpful and convenient [20,22,27–29,31]. Additionally, messaging interventions were associated with increased knowledge of maternal and infant health topics, including awareness of the importance of iron and folic acid supplementation during pregnancy and knowledge about low-birth-weight babies [28]. They also improved communication between healthcare providers and pregnant women, leading to enhanced access to perinatal depression treatments and improved maternal health outcomes [29]. Messaging interventions were found to support maternal and neonatal health during pregnancy and childbirth, reduce home deliveries in favour of health facility deliveries, and increase uptake of antenatal care [21,24,26,28,29]. However, some studies found a lack of significant impact on exclusive breastfeeding rates and the number of follow-up antenatal care visits [23,34]. Despite these variations, the collective evidence suggests that messaging interventions hold promise in positively influencing maternal and infant health outcomes.

It is important to note that several interventions included complementary resources beyond text messaging, such as weekly CHW follow-ups, in-person counselling, transport support systems, non-digital materials, and voice calls [21–23,25,27,30,33]. These additions likely enhanced engagement and impact.

### Barriers and facilitators

**Fig 3:**
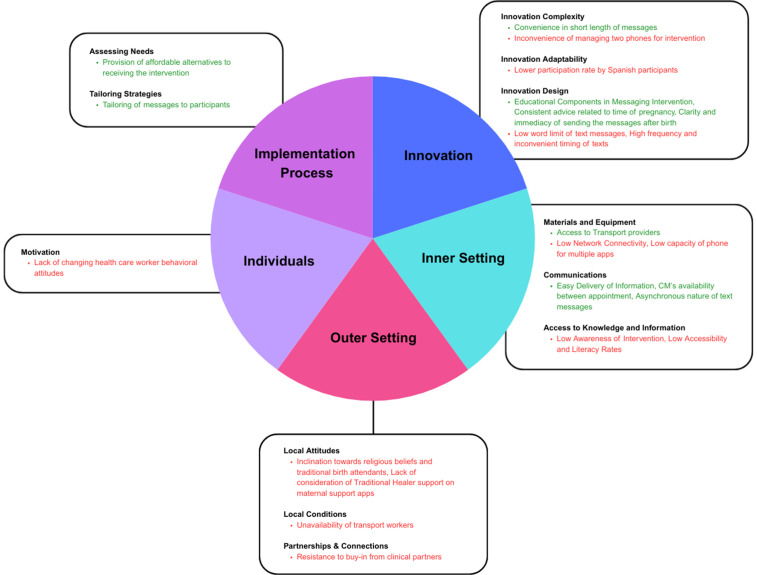
Domains and Constructs captured by Eligible Studies.

A total of 26 barriers and facilitators to the implementation of maternal health messaging interventions were identified across all eligible studies and were categorized using the Consolidated Framework for Implementation Research (CFIR). The detailed extraction of barriers and facilitators, along with their respective excerpts from eligible studies, is included in S4 Table Collapsed Barriers and Facilitators with CFIR. Fig 3 depicts the domains and key constructs with the corresponding barriers and facilitators.

There may be more constructs captured for each domain, however the most critical constructs have been included in the figure. (A) The barriers corresponding with the constructs are noted in red. (B) The facilitators corresponding with the constructs are noted in green.

### Innovation domain

The innovation domain refers to aspects related to the intervention being implemented [38]. The relevant constructs under this domain included innovation adaptability, innovation complexity, and innovation design.

Five out of 15 studies described barriers and facilitators related to the design of the messaging platforms and how it could impact how they valued the information provided [20–22,28,31]. The design of the intervention was noted as a barrier when there were limited word counts restricting detailed health content and messages sent too frequently or at inconvenient times, leading to fatigue and dismissal [28].

One study cited limited adaptability as a barrier, specifically with language being inadequately adapted for Spanish-speaking participants, who had lower participation rates due to the intervention not supporting their native language [27].

Complexity also affected engagement in two studies, particularly when managing intervention tools like multiple phones [27,31]. This logistical issue reduced engagement, as participants sometimes forgot to check messages.

In terms of facilitators for the design of the innovation, clear and relevant messages, with timing and content tailored to pregnancy stages, were appreciated by participants, which helped improve engagement and usefulness [20,22,31].

### Outer setting domain

The outer setting domain refers to external influences on implementation [38]. Constructs relevant under this domain included local attitudes, local conditions, partnerships & connections. In four out of 15 studies, barriers associated with existing perceptions, along with the social and physical environment of the region, affected participants’ willingness and ability to engage with the intervention [21,22,27,30].

In two studies, strong community beliefs led to a preference for traditional birth attendants (TBAs), who are individuals with no formal medical training that assist women during childbirth using traditional practices, over hospital care [21,22]. This reduced engagement with messaging interventions, as TBAs were seen as equally safe as medical care, and the messaging intervention was used only when traditional methods failed [21,22].

Infrastructural conditions in the intervention’s environment affected the effectiveness in another study, where the unavailability of transport workers and limited access to healthcare facilities in rural Nigeria led to many requests for rides being unmet due to a lack of available vehicles at critical times [30].

In another study, buy-in from partners limited its reach and impact as there was resistance from clinical partners, who were hesitant to refer patients to messaging interventions [27].

### Inner setting domain

The inner setting refers to aspects of the setting in which the intervention is being implemented [38]. Relevant constructs under this domain included structural characteristics, relational connections, communications, compatibility, and available resources.

The availability of resources, including funding and infrastructure, was referenced as a barrier or facilitator in five out of 15 studies [21–23,32,33]. Specifically, in four studies, limited resources posed significant barriers, including poor network connectivity, lack of electricity, limited phone storage, and financial constraints preventing access to phones, airtime, or data [21,23,32,33].

Conversely, in two studies, participants lacked access to knowledge and information on how the intervention works or how to actually use the intervention [28,34]. Participants with low digital literacy struggled to use mobile phones effectively, limiting their ability to benefit from text-based messaging interventions [28].

Additionally, lack of compatibility was noted in one study where the messaging platform did not fit well within existing healthcare workflows, leading to inconsistent implementation by midwives and healthcare providers [34]. However, the interventions’ compatibility with the existing workforce was found to be a facilitator in a separate study where the messaging platform reduced the workload of CHWs by streamlining communication [31].

Other facilitators included communication of information and support to participants, which was cited in two out of 15 studies. Participants appreciated having CHWs available between appointments and receiving follow-up calls from midwives, as both forms of direct support provided personalized care and increased their sense of support during pregnancy [29,33].

Available resources were identified as a facilitator in one study, linking women to reliable transport providers, which improved access to healthcare services despite infrastructural challenges [22].

### Individuals domain

The individual’s domain focuses on the traits of the people involved in implementation (CFIR). It included the construct motivation, which was referenced in one out of 15 studies. In the study, there were negative experiences with healthcare providers, such as a perceived lack of concern for patient issues, which discouraged some individuals and impacted their motivation to engage with the intervention [22].

### Implementation process domain

The implementation process domain included the strategies taken to implement the intervention, and included the constructs assessing needs and tailoring strategies, which were present as facilitators in 2 studies. One study found that evaluating participant needs and offering alternative enrollment methods, such as paper registration instead of SMS, increased participation by removing cost barriers [34]. Another study reported that tailoring messages to participants’ pregnancy stages improved the relevance and impact of the information [27]. The constructs reflected in eligible studies are reflected in Table 2.

**Table 2:** Frequency Table of Cited Consolidated Framework for Implementation Research (CFIR) Constructs.

CFIR domains (n = 5) and constructs (n = 67)	Facilitator n (%) of studies	Barrier n (%) of studies
I. Innovation Domain		
*No facilitators or barriers were noted for these constructs related to Innovation Source, Innovation Evidence-Base, Innovation Relative Advantage, Innovation Trialability and Innovation Cost*		
Innovation Complexity	1 (7%)	1 (7%)
Innovation Adaptability	None identified	1 (7%)
Innovation Design	4 (29%)	2 (14%)
II. Outer Setting		
*No facilitators or barriers were noted for these constructs related to Critical Incidents, Policies & Laws, Financing, External Pressures*		
Local Attitudes	None identified	2 (14%)
Local Conditions	None identified	1 (7%)
Partnerships & Connections	None identified	1 (7%)
III. Inner Setting		
*No facilitators or barriers were noted for these constructs related to Structural Characteristics, Culture, Tension for Change, Relative Priority, Incentive Systems, Mission Alignment, Space*		
Relational Connections	1 (7%)	1 (7%)
Communications	3 (21%)	None identified
Compatibility	1 (7%)	1 (7%)
Funding	None identified	2 (14%)
Materials & Equipment	1 (7%)	4 (29%)
Access to Knowledge and Information	None identified	2 (14%)
IV. Individuals		
*No facilitators or barriers were noted for these constructs related to Roles Subdomains, Need, Capability, Opportunity*		
Motivation	None identified	1 (7%)
V. Implementation Process		
*No facilitators or barriers were noted for these constructs related to Teaming, Assessing Context, Planning, Engaging, Doing, Reflecting & Evaluating, Adapting*		
Assessing Needs	1 (7%)	None identified
Tailoring Strategies	1 (7%)	None identified

### Implications of specific CFIR domains and constructs

The presence of constructs like “Infrastructure and Resource Availability” and “Innovation Cost” highlights the critical importance of addressing logistical and economic challenges in rural settings. The lack of affordable access to airtime and phones, combined with limited network coverage, demonstrates how infrastructure can fundamentally limit the success of messaging interventions. The inclusion of “Innovation Design” as a facilitator in some studies demonstrates that when the intervention is tailored to user needs (e.g., clear messaging, easy access), it can enhance engagement and outcomes. On the other hand, domains and constructs like “Financing” or “External Pressure” were not represented, suggesting that issues like funding or external policy pressures were either insignificant in the reviewed studies or not explored. The absence of these constructs could indicate that the interventions were focused more on immediate logistical challenges rather than larger systemic factors, which might be areas for further research. The domains and constructs that are not underrepresented or not represented at all may not be as critical to the mHealth messaging interventions and thus were not captured.

### Emergent Themes

Five overarching themes from the analysis of the results were identified, including: 1. Intervention Accessibility and Content (encompassing the direct delivery of clear, timely text messages and educational components that empower maternal decision-making, while also highlighting barriers such as managing multiple phones, financial constraints, and design limitations related to message length and local language availability), 2. Infrastructure and Resource Availability (reflecting facilitators like linking women with reliable transport providers, yet noting barriers such as poor network connectivity, unavailability of transport workers, and limited phone memory that hinder access to care), 3. Cultural, Religious, and Social Preferences (indicating that in some settings, a preference for traditional birth attendants or healers over digital support can limit intervention uptake), 4. Equitable Engagement and Tailoring (underscoring the benefits of personalized messaging, consistent advice, follow-up calls, and alternative registration methods, while also revealing challenges like lower participation among Spanish-speaking participants and issues arising from shared phone usage), and 5. Healthcare Provider Attitudes (highlighting the importance of clinical partner buy-in and supportive healthcare worker interactions, with barriers including resistance to change and blurred boundaries between professional and personal communication).

## Discussion

This systematic review identified multiple barriers and facilitators to the implementation of messaging platforms to provide maternal support in rural communities from a global perspective across 15 published articles between 2003 and 2025. The analysis captured perspectives from 10 countries, primarily LMICs in Africa and Asia, with the majority being in the United States, South Africa, Kenya, and India. The 14 barriers and 11 facilitators identified in this study are consistent with the literature published on other mHealth interventions over the last two decades [12, 39–40]. This systematic review has implications for current and future message-based interventions targeting maternal health, since the findings suggest that message-based interventions should be tailored to local cultural and infrastructural contexts, ensuring affordability, accessibility, and stakeholder engagement for effectiveness. Future designs must incorporate diverse community input and adaptable features to enhance maternal health outcomes in rural settings. Prior studies on mHealth maternal health programs support this finding and suggest that cost-effective technology, as well as adapting the intervention to both local and national settings, is essential when considering implementation [41].

The consistent success of messaging platforms across 15 studies in improving maternal health outcomes points to their ability to bridge gaps in access, continuity, and personalization of care. Their asynchronous nature allows users to absorb information at their own pace, which may be especially empowering in settings where face-to-face care is limited. The variation in outcomes, such as the limited effect on exclusive breastfeeding, suggests that not all maternal health behaviours are equally responsive to digital prompts [23]. Behaviours that are deeply embedded in social norms or require ongoing physical support may demand more intensive or complementary strategies. This is supported by the eligible studies, where many of the successful interventions had complementary resources, suggesting that messaging alone may be insufficient in low-resource settings, where additional human support and access-enabling components can play a critical role in improving maternal health outcomes. This mirrors broader evidence on mHealth interventions, which have shown promise in addressing maternal mental health, chronic conditions like diabetes, and smoking cessation and substance use during pregnancy [15].

The design of the messaging interventions, particularly the content of the messages, played a crucial role in their success. Pregnant women and mothers valued messaging interventions that were clear, accessible, and educational, which enhanced their sense of agency in making health decisions [20,22,28,31]. The ability to access information flexibly and receive timely reminders supported engagement, particularly for busy mothers navigating competing demands [26,28,31]. This is consistent with research evaluating digital patient education, particularly for maternal health, which finds that interventions, including text and video messages, offer flexibility and a lack of spatial and temporal barriers, which are beneficial for the participant and result in significant changes to outcomes and knowledge levels [42]. Some of these studies relied on self-reported data, which may introduce bias due to social desirability, particularly when evaluating intervention success based on participants’ accounts of how useful or reassuring, they found the messages, how confident they felt using the platform, or how supported they felt during pregnancy [20,22,31,34].

Preferences regarding message length were varied across studies, with participants in one appreciating short, concise messages. In contrast, another study found that the limited length of messages constrained the delivery of more detailed, meaningful health information [28,31].

Findings on messaging frequency were also mixed, with one study reporting that an overload of messages led to fatigue and disengagement, while others found that mothers appreciated regular reminders and prompts for two-way communication [21,22,27,28]. These differences suggest that while consistency is generally beneficial, the timing, tone, length and frequency of messages must be thoughtfully balanced to maintain user interest and participation.

Messaging interventions were also seen as beneficial by health workers, who experienced reduced workloads, indicating system-level efficiency gains [30]. However, poor design, such as requiring separate devices, created usability challenges for some participants [27]. These issues highlight the importance of co-designing interventions with end-users to ensure they integrate seamlessly into daily life, without creating additional burdens. Future research could incorporate mixed-methods approaches that triangulate self-reported experiences with objective indicators, such as clinical outcomes like antenatal care attendance or facility-based deliveries.

Expanding on the CFIR framework can provide valuable insights into the unaddressed domains that could influence the success of messaging interventions for maternal support. For instance, constructs within the Implementation Process domain, such as *Teaming*, *Planning*, and *Engaging,* are crucial for fostering robust community and stakeholder involvement, with cultural leaders potentially aiding local acceptance in places where traditional beliefs and practices play a significant role in healthcare decisions. In the Inner Setting domain, constructs like *Tension for Change* and *Mission Alignment* could uncover whether there is an institutional recognition of the need for change and alignment with the goals of improved maternal care. Furthermore, the Individuals domain, particularly constructs like *Roles Subdomains* and *Capabilities*, is important for understanding the readiness and capacity of those involved. Addressing these overlooked domains not only helps to explain current intervention challenges but also guides the development of future interventions, making them more adaptable, culturally relevant, and sustainable in diverse settings.

Out of the 15 studies, 13 took place in low- and middle-income countries (LMICs). This study focused on rural communities, which should be noted to have a higher magnitude of poverty than urban communities, especially in LMICs [43]. When implementing an intervention, it is important to consider the accessibility factors of the intervention [44]. This was evident in the infrastructural barriers identified in this review, predominantly in LMICs, including poor network connectivity and a lack of available transport drivers, which limited access to both the intervention and nearby healthcare facilities [21,30,32,33]. In the studies in the United States, a high-income country, challenges with infrastructure were not noted but rather focused on how well the technology was integrated with participants’ and workers’ daily lives [27,29].

Additional limitations include low literacy rates in LMICs and rural areas which, compounded with sociodemographic factors, can hinder comprehension and the ability to operate apps and text messages, reducing the effectiveness and uptake of interventions [45,46]. Another accessibility issue that has been noted is the high cost of mobile data, which has been a problem in multiple maternal mHealth interventions, for example, MomConnect in South Africa, where it can act as a significant barrier to access and participation [23,33,41]. To mitigate these challenges, interventions should be designed with user-friendly features, such as voice messages or visual aids, and consideration should be given to providing financial support or subsidies to ensure participants can consistently engage with the program [45]. Some interventions actively addressed equity by reducing cost and access barriers, demonstrating that intentional design choices can enhance inclusivity [22,34]. MomConnect, for example, was able to reduce costs by using unstructured Supplementary Service Data (USSD) technology that can work on very basic smartphones, helping to overcome technological and financial barriers [45]. Global digital health frameworks like the WHO Global strategy on digital health should therefore continue to prioritize investments in the “digital determinants of health,” such as basic infrastructure, affordable connectivity, and mobile accessibility [47].

Tailoring the intervention is crucial for ensuring its relevance and effectiveness across diverse participant needs, cultures, and preferences. Barriers such as language, inconvenient timing and low awareness of the intervention due to practices like phone sharing, often present in LMICs, reflect a disconnect between design and lived experience [27,34,48]. These challenges highlight the importance of culturally grounded approaches developed in partnership with the target population. Meaningful consultation can reveal practical solutions, such as delivering messages in local languages, aligning timing with users’ daily routines, simplifying content, and providing touchpoints like follow-up calls, all of which enhance accessibility, trust, and sustained engagement [27,33]. Educational elements and consistent messaging appear to be effective across multiple studies in both HICs and LMICs, potentially because they build trust, reinforce key health behaviours, and enhance maternal health literacy over time [21,22,27]. Studies support that tailored messages can improve maternal health literacy, support behaviour change, and empower pregnant women to attend antenatal care and deliver in healthcare facilities, both of which are essential for reducing maternal mortality [49].

The relevance and consideration of traditional healers and religious beliefs were not extensively explored in most studies but emerged as a potential area for expansion. The preference for traditional birth attendants (TBAs) and reliance on local healers was noted specifically in rural African settings and suggests that culturally embedded health practices are more trusted and could significantly influence healthcare-seeking behaviours [21,22]. In many rural and remote communities in LMICs, traditional medical systems and birth attendants remain the primary choice for health services [50–52]. For national maternal health policies, particularly in African contexts, the formal inclusion and training of TBAs within rural healthcare systems should be incorporated to enhance their acceptance and effectiveness, since they often have the trust of the community and are believed to have expertise that modern health systems lack [50]. A study in Ghana found that traditional birth attendants are willing to work with formal health services as well, thus integration could help improve acceptability among those who prefer traditional birth attendants [50]. Integrating and training traditional birth attendants (TBAs) in maternal health interventions has been linked to lower perinatal and neonatal deaths and improved connections to formal healthcare, both of which contribute to reducing maternal mortality [51]. Multiple mHealth studies, including those using SMS, have shown that incorporating TBAs has resulted in improvements in the maternal mortality ratio and referral and use of skilled birth attendants [53,54]. This suggests that the incorporation and consideration of traditional birth attendants and healers is important when creating maternal health interventions, particularly in rural communities.

Lastly, community health worker (CHW) and health provider attitudes significantly influenced the success of the mHealth messaging interventions [29]. The challenge of maintaining a work-life balance noted by CHWs suggests that while two-way messaging interventions can enhance participant support, they may also inadvertently create pressures for healthcare providers [29]. This suggests a need to consult the healthcare workers, considering their preferences in the creation of the intervention so that they are not overwhelmed by the demands of constant, informal communication. The lack of buy-in from clinical partners and negative attitudes from healthcare workers noted in 2 studies demonstrate how, without support from key healthcare providers, interventions can struggle to gain traction, limiting referrals and participation [22,27]. Studies have shown that when beneficiaries such as the pregnant women, healthcare workers and community members are involved in co-creation, the interventions are more likely to succeed. For example, involving healthcare providers in the design and implementation process has been found to enhance the acceptability of interventions, ensuring they are appropriately tailored to the needs of both providers and patients [55].

The studies included in this review primarily focused on rural populations in Africa and Asia, with limited representation from rural areas in South America and Europe. This gap limits the global applicability of findings and highlights the need for broader representation of rural communities to better understand contextual differences. While the review demonstrates that messaging interventions can improve maternal health outcomes, it also reveals key areas for improvement such as in technological infrastructure, cultural tailoring, stakeholder engagement and intervention design. Addressing these gaps requires building on current successes while targeting the most pressing behavioural and implementation challenges.

Future research should focus on optimizing interventions for behaviours like exclusive breastfeeding and facility-based deliveries, exploring offline-first apps and voice systems to address literacy and connectivity, and examining how trust, cultural alignment, and community endorsement impact uptake. This will help create interventions that are not only effective but adaptable and scalable across different rural settings.

### Strengths and Limitations

The study provides a comprehensive analysis of maternal mHealth messaging interventions by encompassing a broad range of studies from different regions. The search was not restricted to any countries or languages, so transferable patterns in rural communities are offered globally. However, the search terms, publication dates and number of databases limited the results of this study, as is common with systematic and scoping reviews. If the words identified in the search string were not used or a study was not included in our selected databases, the article would not be captured. Varying definitions of rurality across the studies could also affect comparability of barriers and facilitators across rural communities. We considered articles in all languages; however, the search captured only two articles in languages other than English, which might result in the sample not being representative of all populations. Insights from secondary sources, including reviews, gray literature and program evaluations, were not considered as the inclusion criteria were limited to only primary resources.

Furthermore, choosing the CFIR framework might be seen as a limitation because this framework has been critiqued for not adequately explaining causal mechanisms or the process of change. Despite this, the CFIR framework provided a structured approach to identify and organize barriers and facilitators in mHealth messaging interventions for maternal support, helping to clarify how contextual and design factors shaped implementation in rural settings. However, while there was representation in all 5 domains, only 16 out of 67 constructs were explored, reflecting the limited emphasis on implementation science considerations within existing mHealth messaging intervention studies. Using the CFIR was still valuable as it highlighted areas that were often overlooked, such as individual characteristics, organizational readiness, and strategic planning, which are likely to play an important role in intervention uptake and sustainability. Additionally, it has been effectively used in other similar systematic reviews and helped us situate the findings within the broader scope of implementation research [56–59].

## Conclusion

This review identified several key themes influencing the success of these interventions, including intervention accessibility and content, infrastructure and resource availability; cultural, religious, and social preferences; equitable engagement and tailoring; and stakeholder attitudes. Effective global strategies included providing educational content and integrating support from community health workers, while LMIC-specific strategies emphasized user-friendly, timely messages, local language delivery, flexible access, and low-cost technologies such as USSD. However, barriers, such as financial constraints, poor network connectivity, and cultural preferences for traditional healers can impede the effectiveness.

The majority of the studies demonstrated that messaging platforms could effectively improve maternal health outcomes, such as increasing antenatal visit rates and enhancing maternal knowledge, however two studies noted a lack of significant impact on exclusive breastfeeding and follow-up antenatal care visits. Our findings suggests that mHealth messaging interventions can potentially improve maternal health outcomes but should be interpreted alongside key limitations, including the exclusion of relevant perspectives from non-English-speaking contexts and limited insights into how personal factors and implementation strategies influence results. The findings emphasize the importance of tailoring messaging platform interventions to address the needs of target populations, involving stakeholders in the design process, and incorporating educational components significantly enhance participant engagement and intervention success, particularly in low- and middle-income countries. Policymakers should focus on improving digital infrastructure, reducing data costs, and integrating digital health tools into national health systems to ensure more equitable access to maternal health interventions. Future research should explore complementary technologies such as interactive voice response systems to address literacy and connectivity challenges, and integrate co-design with target populations during intervention development.

## Supporting information

S1 Table PRISMA Checklist. (DOCX)

S2 Table MMAT. (DOCX)

S3 Table Study Characteristics. (DOCX)

S4 Table Collapsed Barriers and Facilitators. (DOCX)

S1 Appendix Search Strings. (DOCX)
