# Supplementary material for: Barriers and facilitators of messaging platforms as a means of maternal support and care in rural communities: A systematic review
Source: PLoS One. 2025 Dec 5;20(12):e0336168. doi: 10.1371/journal.pone.0336168 (PMC12680158; doi:10.1371/journal.pone.0336168)
Supplement: S2 Table — (DOCX) [file pone.0336168.s002.docx]

**S2 MMAT (Mixed Methods Appraisal Tool) for Included Studies**

**Table: MMAT for Included Studies**

| Author/Year | Article Name | Screening Questions (Yes/No/Can’t tell) | | Methodological Quality Criteria (Yes/No/Can’t tell) | |
| --- | --- | --- | --- | --- | --- |
| Cramer, M.E.; Mollard, E.K.; Ford, A.L.; Kupzyk, K.A.; Wilson, F.A. | The feasibility and promise of mobile technology with community health worker reinforcement to reduce rural preterm birth | 1. Are there clear research questions? | Yes | 3. Is there an adequate rationale for using a mixed methods design to address the research question? | Yes |
|  |  |  |  | 4. Are the different components of the study effectively integrated to answer the research question? | Yes |
|  |  | 2. Do the collected data allow to address the research questions? | Yes | 5. Are the outputs of the integration of qualitative and quantitative components adequately interpreted? | Yes |
|  |  |  |  | 6. Are there divergence and inconsistencies between quantitative and qualitative results adequately addressed? | Yes |
|  |  |  |  | 7. Do the different components of the study adhere to the quality criteria of each tradition of the methods involved? | Yes |

| Author/Year | Article Name | Screening Questions (Yes/No/Can’t tell) | | Methodological Quality Criteria (Yes/No/Can’t tell) | |
| --- | --- | --- | --- | --- | --- |
| Datta, S.S.; Ranganathan, P.; Sivakumar, K.S. | A study to assess the feasibility of text messaging service in delivering maternal and child healthcare messages in a rural area of Tamil nadu, India | 1. Are there clear research questions? | Yes | 3. Is there an adequate rationale for using a mixed methods design to address the research question? | Yes |
|  |  |  |  | 4. Are the different components of the study effectively integrated to answer the research question? | Yes |
|  |  | 2. Do the collected data allow to address the research questions? | Yes | 5. Are the outputs of the integration of qualitative and quantitative components adequately interpreted? | Yes |
|  |  |  |  | 6. Are there divergence and inconsistencies between quantitative and qualitative results adequately addressed? | Yes |
|  |  |  |  | 7. Do the different components of the study adhere to the quality criteria of each tradition of the methods involved? | Yes |

| Author/Year | Article Name | Screening Questions (Yes/No/Can’t tell) | | Methodological Quality Criteria (Yes/No/Can’t tell) | |
| --- | --- | --- | --- | --- | --- |
| Bhat, A.; Mao, J.; Unützer, J.; Reed, S.; Unger, J. | Text messaging to support a perinatal collaborative care model for depression: A multi-methods inquiry | 1. Are there clear research questions? | Yes | 3. Is there an adequate rationale for using a mixed methods design to address the research question? | Yes |
|  |  |  |  | 4. Are the different components of the study effectively integrated to answer the research question? | Yes |
|  |  | 2. Do the collected data allow to address the research questions? | Yes | 5. Are the outputs of the integration of qualitative and quantitative components adequately interpreted? | Yes |
|  |  |  |  | 6. Are there divergence and inconsistencies between quantitative and qualitative results adequately addressed? | Yes |
|  |  |  |  | 7. Do the different components of the study adhere to the quality criteria of each tradition of the methods involved? | Yes |

| Author/Year | Article Name | Screening Questions (Yes/No/Can’t tell) | | Methodological Quality Criteria (Yes/No/Can’t tell) | |
| --- | --- | --- | --- | --- | --- |
| *Friday Okonofua1,2,3*, Lorretta Ntoimo1,4, Ermel Johnson5, Issiaka Sombie5, Solanke Ojuolape6, Brian Igboin1,*  *Wilson Imongan1, Chioma Ekwo1, Ogochukwu Udenigwe7, Sanni Yaya7,8, Anne B. Wallis9 and Joy Adeniran1* | Texting for life: a mobile phone application  to connect pregnant women with emergency  transport and obstetric care in rural Nigeria | 1. Are there clear research questions? | Yes | 3. Is there an adequate rationale for using a mixed methods design to address the research question? | Yes |
|  |  |  |  | 4. Are the different components of the study effectively integrated to answer the research question? | Yes |
|  |  | 2. Do the collected data allow to address the research questions? | Yes | 5. Are the outputs of the integration of qualitative and quantitative components adequately interpreted? | Yes |
|  |  |  |  | 6. Are there divergence and inconsistencies between quantitative and qualitative results adequately addressed? | Yes |
|  |  |  |  | 7. Do the different components of the study adhere to the quality criteria of each tradition of the methods involved? | Yes |

| Author/Year | Article Name | Screening Questions (Yes/No/Can’t tell) | | Methodological Quality Criteria (Yes/No/Can’t tell) | |
| --- | --- | --- | --- | --- | --- |
| *Shan Huang1,2* and Mu Li2* | Piloting a mHealth intervention to improve  newborn care awareness among rural  Cambodian mothers: a feasibility study | 1. Are there clear research questions? | Yes | 3. Is there an adequate rationale for using a mixed methods design to address the research question? | Yes |
|  |  |  |  | 4. Are the different components of the study effectively integrated to answer the research question? | Yes |
|  |  | 2. Do the collected data allow to address the research questions? | Yes | 5. Are the outputs of the integration of qualitative and quantitative components adequately interpreted? | Yes |
|  |  |  |  | 6. Are there divergence and inconsistencies between quantitative and qualitative results adequately addressed? | Yes |
|  |  |  |  | 7. Do the different components of the study adhere to the quality criteria of each tradition of the methods involved? | Yes |

| Author/Year | Article Name | Screening Questions (Yes/No/Can’t tell) | | Methodological Quality Criteria (Yes/No/Can’t tell) | |
| --- | --- | --- | --- | --- | --- |
| *Abdul Momin Kazi1,2, MBBS, MPH; Jason-Louis Carmichael3, MPH; Galgallo Waqo Hapanna3, BSC Stat; Patrick*  *Gikaria Wangoo3, MS IT; Sarah Karanja4, MPH; Denis Wanyama4, MBCHB, MPH; Samuel Opondo Muhula4, MSc;*  *Lennie Bazira Kyomuhangi4, MPH/HE; Mores Loolpapit4, MBCHB, MPH; Gilbert Bwire Wangalwa4, MS, MPH;*  *Koki Kinagwi4, MBCHB, MPH; Richard Todd Lester5, MD* | Assessing Mobile Phone Access and Perceptions for  Texting-Based mHealth Interventions Among Expectant Mothers  and Child Caregivers in Remote Regions of Northern Kenya: A  Survey-Based Descriptive Study | 1. Are there clear research questions? | Yes | 3. Is there an adequate rationale for using a mixed methods design to address the research question? | Yes |
|  |  |  |  | 4. Are the different components of the study effectively integrated to answer the research question? | Yes |
|  |  | 2. Do the collected data allow to address the research questions? | Yes | 5. Are the outputs of the integration of qualitative and quantitative components adequately interpreted? | Yes |
|  |  |  |  | 6. Are there divergence and inconsistencies between quantitative and qualitative results adequately addressed? | Yes |
|  |  |  |  | 7. Do the different components of the study adhere to the quality criteria of each tradition of the methods involved? | Yes |

| Author/Year | Article Name | Screening Questions (Yes/No/Can’t tell) | | Methodological Quality Criteria (Yes/No/Can’t tell) | |
| --- | --- | --- | --- | --- | --- |
| *A.S. Laar a,*, E. Bekyieriya a, S. Isang b, B. Baguune* | *Assessment of mobile health technology for*  *maternal and child health services in rural Upper*  *West Region of Ghana* | 1. Are there clear research questions? | Yes | 3. Is there an adequate rationale for using a mixed methods design to address the research question? | Yes |
|  |  |  |  | 4. Are the different components of the study effectively integrated to answer the research question? | Yes |
|  |  | 2. Do the collected data allow to address the research questions? | Yes | 5. Are the outputs of the integration of qualitative and quantitative components adequately interpreted? | Yes |
|  |  |  |  | 6. Are there divergence and inconsistencies between quantitative and qualitative results adequately addressed? | Yes |
|  |  |  |  | 7. Do the different components of the study adhere to the quality criteria of each tradition of the methods involved? | Yes |

| Author/Year | Article Name | Screening Questions (Yes/No/Can’t tell) | | Methodological Quality Criteria (Yes/No/Can’t tell) | |
| --- | --- | --- | --- | --- | --- |
| Watterson, Jessica L.; Castaneda, Diego; Catalani, Caricia | Promoting Antenatal Care Attendance Through a Text Messaging Intervention in Samoa: Quasi-Experimental Study | 1. Are there clear research questions? | Yes | 3. Is there an adequate rationale for using a mixed methods design to address the research question? | Yes |
|  |  |  |  | 4. Are the different components of the study effectively integrated to answer the research question? | Yes |
|  |  | 2. Do the collected data allow to address the research questions? | Yes | 5. Are the outputs of the integration of qualitative and quantitative components adequately interpreted? | Yes |
|  |  |  |  | 6. Are there divergence and inconsistencies between quantitative and qualitative results adequately addressed? | No |
|  |  |  |  | 7. Do the different components of the study adhere to the quality criteria of each tradition of the methods involved? | Yes |

| Author/Year | Article Name | Screening Questions (Yes/No/Can’t tell) | | Methodological Quality Criteria (Yes/No/Can’t tell) | |
| --- | --- | --- | --- | --- | --- |
| Bangal, V.; Somasundaram, K.V.; Thitame, S. | Influence of mobile communication on utilization and outcome of maternal health services in rural area | 1. Are there clear research questions? | Yes | Is randomization appropriately performed? | Yes |
|  |  |  |  | Are the groups comparable at baseline? | Yes |
|  |  | 2. Do the collected data allow to address the research questions? | Yes | Are there complete outcome data? | Yes |
|  |  |  |  | Are outcome assessors blinded to the intervention provided? | No |
|  |  |  |  | Did the participants adhere to the assigned intervention? | Yes |

| Author/Year | Article Name | Screening Questions (Yes/No/Can’t tell) | | Methodological Quality Criteria (Yes/No/Can’t tell) | |
| --- | --- | --- | --- | --- | --- |
| Moleen Zunza ,1 Taryn Young,1 Mark Cotton ,2 Amy Slogrove ,2  Lawrence Mbuagbaw ,1,3 Louise Kuhn,4 Lehana Thabane | Feasibility of using smartphones by village  health workers for pregnancy registration and  effectiveness of mobile phone text messages  on reduction of homebirths in rural Uganda | 1. Are there clear research questions? | Yes | 3. Are the participants representative of the target population? | Yes |
|  |  |  |  | 4. Are measurements appropriate regarding both the outcome and intervention (or exposure)? | Yes |
|  |  | 2. Do the collected data allow to address the research questions? | Yes | 5. Are there complete outcome data? | Yes |
|  |  |  |  | 6. Are the confounders accounted for in the design and analysis? | Yes |
|  |  |  |  | 7. During the study period, is the intervention administered (or exposure occurred) as intended? | Yes |

| Author/Year | Article Name | Screening Questions (Yes/No/Can’t tell) | | Methodological Quality Criteria (Yes/No/Can’t tell) | |
| --- | --- | --- | --- | --- | --- |
| *Gershim Asiki1,2,3*, Robert Newton2,4, Leonard Kibirige2, Anatoli Kamali2, Lena Marions5,*  *Lars Smedman* | Feasibility of using smartphones by village  health workers for pregnancy registration and  effectiveness of mobile phone text messages  on reduction of homebirths in rural Uganda | 1. Are there clear research questions? | Yes | 3. Are the participants representative of the target population? | Yes |
|  |  |  |  | 4. Are measurements appropriate regarding both the outcome and intervention (or exposure)? | Yes |
|  |  | 2. Do the collected data allow to address the research questions? | Yes | 5. Are there complete outcome data? | Yes |
|  |  |  |  | 6. Are the confounders accounted for in the design and analysis? | Can’t tell |
|  |  |  |  | 7. During the study period, is the intervention administered (or exposure occurred) as intended? | Yes |

| Author/Year | Article Name | Screening Questions (Yes/No/Can’t tell) | | Methodological Quality Criteria (Yes/No/Can’t tell) | |
| --- | --- | --- | --- | --- | --- |
| Maricianah Onono,* Gladys Ombonya Odhiambo, Ouma Congo, Lawrence Wandei Waguma, Titus Serem,  Mildred Anyango Owenga, and Pauline Wekesa | Narratives of Women Using a 24-Hour Ride-Hailing Transport System to Increase Access and  Utilization of Maternal and Newborn Health Services in Rural Western Kenya: A Qualitative Study | 1. Was there a clear statement of the aim of the research? | Yes | 3. Is the qualitative approach appropriate to answer the research question? | Yes |
|  |  |  |  | 4. Are the qualitative data collection methods adequate to address the research question? | Yes |
|  |  | 2. Do the collected data allow to address the research questions? | Yes | 5. Are the findings adequately derived from the data? | Yes |
|  |  |  |  | 6. Is the interpretation of results sufficiently substantiated by data? | Yes |
|  |  |  |  | 7. Is there coherence between qualitative data sources, collection, analysis and interpretation? | Yes |

| Author/Year | Article Name | Screening Questions (Yes/No/Can’t tell) | | Methodological Quality Criteria (Yes/No/Can’t tell) | |
| --- | --- | --- | --- | --- | --- |
| *Livhuwani Muthelo 1,* , Masenyani Oupa Mbombi 1 , Mamare Adelaide Bopape 1 , Tebogo M. Mothiba 1 ,*  *Melissa Densmore 2, Alastair van Heerden 3 , Shane A. Norris 4 , Nervo Verdezoto Dias 5, Paula Griffiths 6*  *and Nicola Mackintosh 7* | Reflections on Digital Maternal and Child Health Support for  Mothers and Community HealthWorkers in Rural Areas of  Limpopo Province, South Africa | 1. Was there a clear statement of the aim of the research? | Yes | 3. Is the qualitative approach appropriate to answer the research question? | Yes |
|  |  |  |  | 4. Are the qualitative data collection methods adequate to address the research question? | Yes |
|  |  | 2. Do the collected data allow to address the research questions? | Yes | 5. Are the findings adequately derived from the data? | Yes |
|  |  |  |  | 6. Is the interpretation of results sufficiently substantiated by data? | Yes |
|  |  |  |  | 7. Is there coherence between qualitative data sources, collection, analysis and interpretation? | Yes |

| Author/Year | Article Name | Screening Questions (Yes/No/Can’t tell) | | Methodological Quality Criteria (Yes/No/Can’t tell) | |
| --- | --- | --- | --- | --- | --- |
| *B. McBride1, J.D. O’Neil2, Trinh T. Hue3, R. Eni1, C. Vu Nguyen3, L.T. Nguyen* | Improving health equity for ethnic minority women in Thai  Nguyen, Vietnam: qualitative results from an mHealth  intervention targeting maternal and infant health service  access | 1. Was there a clear statement of the aim of the research? | Yes | 3. Is the qualitative approach appropriate to answer the research question? | Yes |
|  |  |  |  | 4. Are the qualitative data collection methods adequate to address the research question? | Yes |
|  |  | 2. Do the collected data allow to address the research questions? | Yes | 5. Are the findings adequately derived from the data? | Yes |
|  |  |  |  | 6. Is the interpretation of results sufficiently substantiated by data? | Yes |
|  |  |  |  | 7. Is there coherence between qualitative data sources, collection, analysis and interpretation? | Yes |

| Author/Year | Article Name | Screening Questions (Yes/No/Can’t tell) | | Methodological Quality Criteria (Yes/No/Can’t tell) | |
| --- | --- | --- | --- | --- | --- |
| Gitonga et al., 2021 | Influence of short message service reminders on  utilisation of focused antenatal care among women in rural Kenya: a randomised controlled trial | 1. Are there clear research questions? | Yes | 3. Is randomization appropriately performed? | Yes |
|  |  |  |  | 4. Are the groups comparable at baseline? | Yes |
|  |  | 2. Do the collected data allow to address the research questions? | Yes | 5. Are there complete outcome data? | Yes |
|  |  |  |  | 6. Are outcome assessors blinded to the intervention provided? | Yes |
|  |  |  |  | 7. Did the participants adhere to the assigned intervention? | Yes |
