# Supplementary material for: Barriers and facilitators of messaging platforms as a means of maternal support and care in rural communities: A systematic review
Source: PLoS One. 2025 Dec 5;20(12):e0336168. doi: 10.1371/journal.pone.0336168 (PMC12680158; doi:10.1371/journal.pone.0336168)
Supplement: S3 Table — (DOCX) [file pone.0336168.s003.docx]

**S3. Table: Study Characteristics**

| **Study characteristics** | | | | | | | | |
| --- | --- | --- | --- | --- | --- | --- | --- | --- |
| # | Study title | Author names | Year of publication | Country | Type of study | Population/subgroup | Study objective | Evidence of Impact |
| 1 | The feasibility and promise of mobile technology with community health worker reinforcement to reduce rural preterm birth | Cramer, M.E.; Mollard, E.K.; Ford, A.L.; Kupzyk, K.A.; Wilson, F.A. | 2018 | United Stated | Mixed | Rural community members | The primary aim of this pilot study was to assess the feasibility of a tailored smartphone platform intervention (hereinafter re‐ ferred to as prenatal technology platform [PTP]) combined with CHW reinforcement among rural pregnant women. | Fifty‐one participants completed the CSQ‐8 (N = 16 control and N = 35 intervention). Intervention participants were satisfied and scored higher on the CSQ‐8 (M = 3.59, SD = 0.3) than the control (M = 3.22, SD = 0.7).  Overall, participants were highly engaged and receptive to the PTP. There were 241 chats among 41 participants. Most were initiated by participants (i.e., in‐ coming) during the first few months following enrollment. |
| 2 | A study to assess the feasibility of text messaging service in delivering maternal and child healthcare messages in a rural area of Tamil nadu, India | Datta, S.S.; Ranganathan, P.; Sivakumar, K.S. | 2014 | India | Mixed | Rural population from Vellore district, Tamil Nadu | To evaluate whether mobile Text Messaging Service is a feasible mode of raising knowledge regarding maternal and child health (MCH) and to explore issues related to mobile text messages as a mode of health education. | Significant increases in knowledge related to MCH care were observed after texts were sent to study participants. After receiving text messages, more than half (67, 55.83 per cent) knew the minimum number of iron folic acid tablets to be consumed by a pregnant mother, as compared to 26 (21.67 per cent) individuals before receiving text messages (P < 0.05, 95 per cent CI:0.21–0.46). Similarly, 52 (43.33 per cent) individuals knew about low birth weight babies after receiving text messages, as compared to 24 (20 per cent) individuals before receiving texts (P < 0.05, 95 per cent CI:0.12–0.35). Knowledge of maternal health care improved considerably, relative to that about child health care (Table 2). |
| 3 | Text messaging to support a perinatal collaborative care model for depression: A multi-methods inquiry | Bhat, A.; Mao, J.; Unützer, J.; Reed, S.; Unger, J. | 2018 | United States | Mixed | Breede Valley rural community | Mental health care integrated into obstetric settings improves access to perinatal depression treatments. Digital interactions such as text messaging between patient and provider can further improve access. We describe the use of text messaging within a perinatal Collaborative Care(CC) program, and explore the association of text messaging content with perinatal depression outcomes. | Seventeen women completed the survey at the end of the study. All of them reported using text messages to communicate with their CM. Ninety four percent (n=16) of them found it helpful. In a “choose all that apply” question regarding usage, a majority of the women reported using text messaging most frequently for scheduling or rescheduling appointments (88%, n=15). Patients also used text messaging for questions about problem solving therapy homework (24%, n = 4), questions about depression or anxiety symptoms (24%, n= 4), questions about medications (12%, n= 2) and questions about physical symptoms (12%, n =2). Ninety four percent (n=16) of the women surveyed felt comfortable discussing personal information via text messaging, and 88% (n=15) said they felt comfortable sharing the content of the text messages with their partner or spouse. |
| 4 | Evaluating interactive weekly mobile  phone text messaging plus motivational  interviewing for breastfeeding  promotion among women living with  HIV, giving normal birth at a primary  healthcare facility in South Africa: a  feasibility randomised controlled trial | Moleen Zunza ,1 Taryn Young,1 Mark Cotton ,2 Amy Slogrove ,2  Lawrence Mbuagbaw ,1,3 Louise Kuhn,4 Lehana Thabane | 2023 | South Africa | Quantitative | Primary healthcare facility, serving a rural community | We assessed the feasibility of an appropriately  powered randomised trial by evaluating whether  participants could be recruited and retained, and sought  preliminary information on exclusive breastfeeding rates.  Setting Primary healthcare facility, serving a rural community. | Exclusive breastfeeding rates remained relatively high across both groups through week 24 (table 3). Although the intervention group had higher rates of exclusive breast feeding at week 24 than the control group (77.8% vs 55.6%), this difference was not significant: rate difference 22.2% (95% CI −20.1% to 64.5%). All the 18 mothers who completed study follow-up at week 24 were breast feeding (either exclusively in 12 of cases or non-exclusively). |
| 5 | Narratives of Women Using a 24-Hour Ride-Hailing Transport System to Increase Access and  Utilization of Maternal and Newborn Health Services in Rural Western Kenya: A Qualitative Study | Maricianah Onono,* Gladys Ombonya Odhiambo, Ouma Congo, Lawrence Wandei Waguma, Titus Serem,  Mildred Anyango Owenga, and Pauline Wekesa | 2019 | Kenya | Qualitative | Rural Western Kenya | The primary purpose of the qualitative analysis herein was to explore the ways in which pregnant and postnatal women made decisions regarding care-seeking for pregnancy and childbirth services, the processes of getting care from home to the hospital as well as their perceptions on how the MAccess intervention affected their pregnancy and childbirth careseeking and utilization experience. | The MAccess innovation was highly acceptable to women throughout preg- nancy and childbirth and helped them navigate the complex and layered individual, infrastructural, and health system factors that put them at risk of adverse maternal and newborn outcomes. |
| 6 | Texting for life: a mobile phone application  to connect pregnant women with emergency  transport and obstetric care in rural Nigeria | Friday Okonofua1,2,3*, Lorretta Ntoimo1,4, Ermel Johnson5, Issiaka Sombie5, Solanke Ojuolape6, Brian Igboin1,  Wilson Imongan1, Chioma Ekwo1, Ogochukwu Udenigwe7, Sanni Yaya7,8, Anne B. Wallis9 and Joy Adeniran1 | 2023 | Nigeria | Mixed | Rural Local Government Areas  (LGAs) of Edo State, in southern Nigeria | The objective of this paper is to describe the design and implementation of this technology and to reflect on its potentials for reducing the rate of maternal morbidity  and mortality in rural communities. | Of those reporting complications to the platform, 51 (91.1%) were transported by registered taxis to the PHCs.  The common complications reported included bleeding,  preterm labour, severe lower abdominal pain, and severe back pain among others. The reasons for five women not being transported included “taxi not available (on other  duties) at the time” (4), while one woman delivered at home before the taxi arrived. Among the 51 women transported to the PHCs, 46  were successfully treated, while five were referred and transferred by the same taxi to a referral hospital. No maternal deaths were recorded among the women  registered under the platform during the period, although four perinatal deaths occurred. Among the perinatal deaths, one was a macerated stillbirth in a woman who had foetal death in utero; two were stillbirths following labour at a secondary care hospital, while the remaining  death was an early neonatal death due to preterm delivery. |
| 7 | Reflections on Digital Maternal and Child Health Support for  Mothers and Community HealthWorkers in Rural Areas of  Limpopo Province, South Africa | Livhuwani Muthelo 1,* , Masenyani Oupa Mbombi 1 , Mamare Adelaide Bopape 1 , Tebogo M. Mothiba 1 ,  Melissa Densmore 2, Alastair van Heerden 3 , Shane A. Norris 4 , Nervo Verdezoto Dias 5, Paula Griffiths 6  and Nicola Mackintosh 7 | 2023 | South Africa | Qualitative | rural areas of Limpopo Province in South Africa | This study aims to explore the perspectives of mothers, community health workers, and community leaders on maternal and child health digital support in rural,disadvantaged areas of South Africa. | Mothers, community leaders, and health workers reflected on the use of existing maternal and child health apps such as Pregnancy+ and MoMConnect. Digital maternal and health apps (Pregnancy+ and MoMConnect) have an essential role in supporting MCH services such as receiving constant advice messages about pregnancy and fetal development, clinic and follow-up visits, and child care and development after birth. Study findings suggest the supportable use of MCH apps by mothers, community leaders, and healthcare workers in rural areas to ensure that all pregnant women and mothers enjoy the benefits brought by the two apps. |
| 8 | Improving health equity for ethnic minority women in Thai  Nguyen, Vietnam: qualitative results from an mHealth  intervention targeting maternal and infant health service  access | B. McBride1, J.D. O’Neil2, Trinh T. Hue3, R. Eni1, C. Vu Nguyen3, L.T. Nguyen | 2018 | Vietnam | Qualitative | Rural Thai Nguyen Province | Through its research design, this intervention (the mMom project) aimed  to utilize BCC, emphasize interaction between women and CHWs, and evaluate women’s confidence and readiness to access health care. | Acceptability: Participants were unanimous in their satisfaction with receiving regular, timely MNCH information via their mobile phones, asserting that this medium was highly convenient and allowed information to be saved and shared. When asked, most participants reported willingness to pay a nominal fee for the service, suggesting high perceived value of the intervention despite economic constraints. Further, most expressed desire to receive messages during future pregnancies. |
| 9 | Piloting a mHealth intervention to improve  newborn care awareness among rural  Cambodian mothers: a feasibility study | Shan Huang1,2* and Mu Li2 | 2017 | Cambodia | Mixed | Kampong Chhnang rural area | We piloted a mHealth project in this rural province to ascertain the feasibility of a scalable program based on a contextual framework using an Interactive Voice Response (IVR) technology. The aim of this paper is to document how we designed the pilot mHealth project and results of whether or not it would be feasible to use a mHealth approach as a means for promoting timely information to improve mothers’ awareness regarding neonatal health in rural Cambodia. | The results indicate that acceptability was high with all mothers listening to at least four of the seven messages. Sixty-one percent mothers surveyed report that they told others about the service, 71% would recommend the service to other mothers and 63% reported sharing the information they heard with others. The results from our FGD with the health workers also responded well to the program. They report that the program was a good way of connecting with mothers, as one midwife said ‘This (initiative) makes mothers more aware of how to take care of (their) babies’. Our quantitative results indicate that 85% of the mothers surveyed listened to some messages not just once but several times, using the option in the IVR to repeat again, allowing the messages to be heard by other members of the household such as their husbands (n = 87), mothers (n = 67), siblings and older children (n = 23). The FGD results also showed that some first-time mothers said they had ‘shared the messages for other members of the household to listen to’. |
| 10 | Assessing Mobile Phone Access and Perceptions for  Texting-Based mHealth Interventions Among Expectant Mothers  and Child Caregivers in Remote Regions of Northern Kenya: A  Survey-Based Descriptive Study | Abdul Momin Kazi, Jason-Louis Carmichael, Galgallo Waqo Hapanna, Patrick Gikaria Wangoo, Sarah Karanja, Denis Wanyama, Samuel Opondo Muhula, Lennie Bazira Kyomuhangi, Mores Loolpapit, Gilbert Bwire Wangalwa, Koki Kinagwi, Richard Todd Lester | 2017 | Kenya | Mixed | 6 government health facilities in Isiolo, Marsabit, and Samburu counties in remote and northern arid lands (NAL). | Our objectives were to examine region-based differences in mobile phone access and mHealth perceptions among visitors to antenatal and routine immunizations clinics in 8 regions of northern Kenya. | Despite regional differences, there was no significant difference in the proportion of participants from NAL and central highlands, who indicated that they would like to receive a weekly SMS text message from their health care provider (95.0% vs 97.0%; P=.52). Overall, more than 91.6% (230/251) of participants who had access to a mobile phone indicated that they would like to receive a weekly SMS text message from their health care provider. Of these, 51.2% (124/242) preferred SMS text message as their mode of communication, whereas 48.8% (118/242) favored a phone call.  92.0% (230/250) of participants who had access to a telephone said that they would like to receive a weekly SMS text message from their health care provider. Most phone users already spent the equivalent of 626 SMS text messages on mobile credit for personal use. |
| 11 | Feasibility of using smartphones by village  health workers for pregnancy registration and  effectiveness of mobile phone text messages  on reduction of homebirths in rural Uganda | Gershim Asiki1,2,3*, Robert Newton2,4, Leonard Kibirige2, Anatoli Kamali2, Lena Marions5,  Lars Smedman | 2018 | Uganda | Quantitative | Kalungu, Uganda | We implemented a community based pragmatic intervention assess the feasibility of using smartphones for pregnancy registration  and relaying standard health text messages to pregnant women via village health workers  and evaluated the effect of the intervention on reduction of home deliveries. The ultimate  goal of this intervention was to contribute information to future trials for improvement of  child survival in the critical period surrounding birth. | Of the 525 pregnant women, 442 (84.2%) reported delivering in a health facility, 18 (3.4%) delivered at home under traditional birth attendant supervision, 54 (10.3%) were helped by a relative or a friend at home and 11 (2.1%) delivered alone. Fig 2 represents the change in incidence of deliveries in control and intervention villages and reveals substantial reduction in home deliveries after introduction of SMS in January 2015, with some months having no home births in the intervention villages. The intra-cluster correlation coefficient for the outcome was computed to be 0.25. Village health workers were able to deliver the messages to 60% of pregnant women registered in the study and this was associated with a substantial reduction in home births. |
| 12 | Assessment of mobile health technology for  maternal and child health services in rural Upper  West Region of Ghana | A.S. Laar a,*, E. Bekyieriya a, S. Isang b, B. Baguune | 2019 | Ghana | Mixed | T4MCH intervention districts  (WaWest, Wa East, and Jirapa) of the Savana Signatures in the UWR of Ghana | This study assessed Savana Signatures and Global Affairs of Canada Technology for Maternal and Child Health (T4MCH) in  selected districts in the UWR of Ghana to understand the enablers  and barriers for using the technology to provide and use healthcare services | All the women who received follow-up mobile calls (100%) and the majority (98.2%) of those who did not receive follow-up calls expressed high optimism toward receiving future follow-up mobile reminders from health workers. Some of the participants equally expressed high acceptability levels as demonstrated in the sentiments below: "... follow-up phone reminders by health providers will make us to be in constant contact with the nurses for information on our health without being physically present at the health facility. I think this way will definitely reduce pressure of always having to travel to the health facility for certain information which we can equally sit at our homes comfortably and get from the nurses (FGDdPregnant women and lactating mothersc all districts)." |
| 13 | Influence of mobile communication on utilization and outcome of maternal health services in rural area | Bangal, V.; Somasundaram, K.V.; Thitame, S. | 2018 | India | Quantitative | Rural Population in Loni, India | Present study was carried out to assess the influence of mobile communication between health facility and pregnant women on utilization and outcome of maternal health services. | Women in the intervention group had significantly higher number of antenatal visits as compared to the control group. (Table 1) They visited clinic more often than prescribed minimum schedule of visits. Women from intervention group (81%) had consumed iron and calcium tablets for more than three months, after enrolment in the study, as compared to women in control group (69%).The immunization coverage of tetanus toxoid (Inj TT) among pregnant women was 97.00% in intervention group ,as against 87.00% in control group. Satisfactory weight gain (>10KG) was observed in 35.00% of pregnant women from intervention group as against 25.00% in control group. Ultrasound examination for anomaly scan was performed in 93.00%women from intervention group as compared to 79.00% from control group. |
| 14 | Promoting Antenatal Care Attendance Through a Text Messaging Intervention in Samoa: Quasi-Experimental Study | Watterson, Jessica L.; Castaneda, Diego; Catalani, Caricia | 2020 | Samoa | Mixed | Upolu, Samoa | Therefore, this study explores whether this intervention can be effective in the Samoan context, contributing to a more nuanced understanding of how the setting and implementation factors might affect the outcomes of a pregnancy SMS text messaging program. | Using the intention-to-treat principle, women registering at intervention clinics attended, on average, only 2.2 follow-up visits, as compared with 2.6 in the comparison group (P=.01). Similarly, in the per-protocol analysis, women receiving the intervention attended only 2.1 follow-up visits on average, compared with 2.5 visits in the comparison group (P<.001). These unadjusted comparisons are presented in Table 3. Contrary to hypothesis 1, the negative binomial regression analyses (Table 4) showed that women in the intervention group attended 13% (intention-to-treat) to 15% (per-protocol) fewer follow-up ANC visits than women in the comparison group, controlling for all covariates. The interaction term between younger women (defined as under 25 years old) and receiving the intervention in the subsequent regression model was not significant (P=.30), suggesting that the effect of the intervention on ANC attendance was similar across age groups (results not shown in table). Therefore, support was not found for hypothesis 2. |
| 15 | Influence of short message service reminders on  utilisation of focused antenatal care among women in rural Kenya: a randomised controlled trial | Eliphas Gitonga  Jackim Nyamari  Peterson Warutere  Anthony Wanyoro | 2021 | Kenya | Randomized control trial | Rural Pregnant women | This study  aimed to determine the influence of short message service reminder texts on uptake of  focused antenatal care. T | Results indicate that the intervention group had higher uptake of focused  antenatal care; 75.4% compared to 10.2% in the control group. There was a significant  association between the reminders and uptake of focused antenatal care (P<0.001). Binary  logistic regression showed uptake of focused antenatal care increased by 27 times (P<0.001)  for those who received a short message service reminder compared to the control group. |
