# Supplementary material for: Barriers and facilitators of messaging platforms as a means of maternal support and care in rural communities: A systematic review
Source: PLoS One. 2025 Dec 5;20(12):e0336168. doi: 10.1371/journal.pone.0336168 (PMC12680158; doi:10.1371/journal.pone.0336168)
Supplement: S4 Table — (DOCX) [file pone.0336168.s004.docx]

**S4: Collapsed Barriers and Facilitators Tables with CFIR Table 1. Facilitators**

|  | **Facilitator** | **Studies** | **Excerpts** | **Country** | **CFIR** |
| --- | --- | --- | --- | --- | --- |
| 1 | Tailoring of messages to participants | Cramer et al., 2018 | Participants stated that they valued the information provided through the PTP (e.g., “the personalized text messages were very helpful, and I learned different info that I had no clue about”, “the advice and text messages were a good reminder on topics that you may not think of”, and “learned about nutrition and resources that I was able to share with other moms”). Participants liked the personalized aspects of the program and individualized contacts with the CHW via technology (e.g., “Knowing that I could call/text her,” “[I liked] personal contact with the representative”). Several commented on the ease of the PTP (e.g., “very easy to follow,” “I liked getting texts every week,” “it was convenient to get the reminders”) | US | Implementation Process  Construct : Tailoring Strategies |
| 2 | Easy Delivery of Information | Datta et al., 2014 | "The direct delivery of information"  "Text messages can be delivered to masses without much complexity"  "The opportunity to issue reminders" | India | Inner Setting  Construct: Communications |
| 3 | CM’s availability between **appointment** | Bhat et al., 2018 | Patients, on the other hand, greatly appreciated the CM’s availability for text messages in between appointments and after. | US | Inner Setting  Construct: Communications |
| 4 | **A**synchronous nature of text messages | Bhat et al., 2018 | Patients found availability between appointments and asynchronous nature of text messages useful as they had multiple competing priorities like childcare. |  | Inner Setting  Construct: Communications |
| 4 | Educational Components in Messaging Intervention | Onono et al., 2019 | Bidirectional text messages such as those in the MAccess intervention are an innovative way to expand educational opportunities for women regarding birth preparedness. In this study, it was clear that the bidirectional text messages influenced decision-making process for the mothers by increasing their knowledge on danger signs, individual birth plans, possible complications, and immediate post-delivery neonatal care. This is demonstrated in the quotes in the following paragraph, where women reveal that the MAccess intervention helped them alleviate barriers to decision-making and birth preparedness. I have learnt about the many ways of taking care of a child during pregnancy and after delivery. I also learnt of the things to do while pregnant so as to have a healthy pregnancy as well as safe delivery and the benefits of going to the clinic. I have also learnt about having a birth plan and saving money before delivery. I have also learnt through the text messages about the importance of vaccination. They would tell you all the things you need to do during and after pregnancy.The messages that helped me most were: the ones that encouraged me to go to the clinic, the ones about the kind of foods I was to eat and those reminding me to do some exercises. They also gave me helpful answers to the questions I asked them. I used to feel abdominal pain and they told me to lie on back and after a short while the pain went away. They were very helpful. I also asked what to do in case I do not feel the baby play for three consecutive days and they advised me to go to the hospital. (Atieno, 33 year old with 5 living children) | Kenya | Innovation  Construct: Innovation Design |
|  |  | McBride et al., 2018 | All participants reported that the BCC and reminder messages provided valuable MNCH information, which promoted their knowledge of pregnancy and newborn care: For my first child [pregnancy], I was not aware of many  things: for example, during the first month of pregnancy, I have to take an iron supplement; at 6 and a half months, I  have to take calcium […] but for this second child, the program reminds me, so I know how to take care of myself  and my baby.—P1, focus group, Binh Yen Intervention Commune  When seeing the messages, I feel that the program is very beneficial, good for pregnant women, children; good for moms with small babies. The program has a lot of useful information that I have learned from to take care of my  baby properly.—27-Year-old Tay woman with one 5-year-old and one 6-month baby | Vietnam | Innovation  Construct: Innovation Design |
| 5 | Access to Transport providers | Onono et al., 2019 | Although the MAccess did not improve road infrastructure, it linked women with transport providers who were reliable, given the rough terrain, odd hours of labor, and harsh weather. Women reported that the transport providers treated them with care when they were transporting them to the hospitals and they also assisted them to locate the health-care workers once they arrived in the hospitals. Motorized transportation such as motorcycles in this study has been shown to reduce referral delays for labor and delivery by up to 76%...MAccess riders respond very quickly. I was carried by different riders and they were very careful, they would ride. smoothly too. The normal transport riders are very rough. I remember the one who took me to hospital that morning when I was in labor for the first time, he was very rough. He would throw me up and down over the bumps. The one who took me during the second pregnancy (MAccess rider) was very careful. I remember that it was not only dark but it had also rained. Could it have been a normal transport rider, he could have not even agreed to come because it was very muddy | Kenya | Inner Setting  Construct: Available Resources (Materials & Equipment) |
| 6 | Reduction of CHW workload | Okonofuna et al., 2023 | Most CHWs expressed that the mMom intervention had reduced their workloads and improved their working efficiency. Many noted that because mMom was providing timely information to pregnant women, they had to spend less time attending to group counselling.  In the past, we have had to organize communication events a few times per year for pregnant women and women with small kids. But now that the SMS system is in operation, we don’t have to give group counseling.— CHW 1, in focus group discussion | Rural Nigeria | Inner Setting  Construct:  Compatibility |
| 7 | Consistent advice related to time of pregnancy | Muthelo et al., 2023 | CHW 3 added by saying “it also helps when you are pregnant you know that at this time  how far are you and when should you expect to give birth and as a mother you start to prepare for  birth and buy clothing. It provides us with more information that we don’t know”.  Mother 4 added “since I have been pregnant I have been getting those messages from momconnect advising me about the Pregnancy . . . After I gave birth I received a message . . . about going to the clinic for immunization and growth monitoring even now my child is one year, am still getting those messages”. | Rural South Africa | Innovation  Construct: Innovation Design |
| 8 | Convenience in short length of messages | Huang et al., 2017 | Further, 96% of respondents reported the length of the messages (60 to 90 s) was appropriate and all respondents reported that the intervention was helpful for them when looking after their newborn. Additionally, 45% also reported learning something new from listening to the messages. The majority of respondents (96%) report that they would like to see the service extended, 44% of which suggested a service extension of at least 12 months. | Cambodia | Innovation  Construct: Innovation Complexity |
| 9 | Clarity and immediacy of sending the messages after birth | Huang et al., 2017 | Discussion during the FGDs included one first-time mother mentioning that ‘At anytime (when I was listening to the message) the line was clear. The timing was convenient for me because normally mothers with new babies stay home after birth and do not go anywhere’. | Cambodia | Innovation  Construct: Innovation Design |
| 10 | Support from follow up calls | Laar et al., 2019 | For the 10.2% of the women who received follow-up call reminders,  discussions were on issues such as home visits,family planning, dangerous practices after delivery, early postnatal care, newborn care, when to visit the health facility, information on danger signs, and reminders about gestation birth date, the need for delivery in a facility, and others. All the women who had previous contact with health providers see the follow-up reminders as important and helpful. They also expressed satisfaction in the information they received from health providers as indicated in Table 4. These sentiments are also affirmed by the FGD participants in the quotes below:  They have been calling me to ask about my pregnancy. I always feel very happy and relieved whenever they call to ask about my health (FGDdPregnant womandWa West).  I felt privileged to be called by a midwife to find out about my health and my baby … (FGDdLactating motherdJirapa).  …. sometimes we have health problems but because of the distance to the health facility we are not able to go…. Now that they are  going to be contacting us through phones, it is good (FGDdPregnant women and lactating mothers dall districts). | Ghana | Inner Setting  Construct: Relational Connections |
| 11 | Provision of affordable alternatives to receiving the intervention | Watterson et al., 2020 | One of the key facilitators identified was offering the option for women to enroll in the message by paper during their ANC visit, rather than requiring them to send an SMS text message to enroll. Many mobile messaging platforms enroll participants by having them send a short code to a phone number. However, this can cost the participant’s mobile phone credit to send a message. All but one of the participants in this study chose to enroll by paper, suggesting that it was the preferred enrollment option in this population. | Samoa | Implementation Process  Construct:  Assessing Needs |

**Table 2. Barriers**

|  | **Barriers** | **Studies** | **Excerpts** | Country | CFIR |
| --- | --- | --- | --- | --- | --- |
| 1 | Lower participation rate by Spanish participants (Language barrier) | Cramer et al., 2018 | English‐ speaking participants were more engaged than Spanish‐speaking only participants; however, anecdotally, the CHW believed the lower participation rate was because most Spanish‐only speaking participants worked at the local meat packing plant where phone use was discouraged. | US | Innovation  Construct:  Innovation Adaptability |
| 2 | Inconvenience of managing two phones for intervention | Cramer et al., 2018 | We learned during the study that most participants (86%) already owned a smartphone, had unlimited texting (86%), and at‐home Wi‐Fi (65%). Thus, participants said that it had been inconvenient to carry two phones (both theirs and the study phone). Participants sometimes forgot to carry the study phone and check intervention messages. Some of the provided phones transmitted errant spam messages from old phone numbers causing participants to turn off their phones for a week or more | US | Innovation  Construct:  Innovation Complexity |
| 3 | Resistance to buy-in from clinical partners | Cramer et al., 2018 | Issues with clinical partners included getting provider buy in and changes in clinical practice after initiation of the study. Although we had the support of the community advisory board, and many clinical providers not all providers were motivated to refer patients. | US | Outer Setting  Construct: Partnerships & Connections |
| 4 | Low Accessibility and Literacy Rates | Datta et al., 2014 | The absence of the Tamil font (local language) in many handset models.  Illiterate individuals being unable to read text messages.  Limited mobile phone literacy among the general population in the study area. | India | Inner Setting  Construct:  Access to knowledge & Information |
| 5 | Low word limit of text messages | Datta et al., 2014 | Text limits that can prevent the receipt of lengthy messages as many mobile handsets provide up to 90 alphabet limits for text messages.  The possibility of dissemination of incorrect message(s) from the messenger or incorrect interpretation by the recipient(s). | India | Innovation  Construct:  Innovation Design |
| 6 | High frequency and inconvenient timing of texts | Datta et al., 2014 | The opportunity to ignore messages during busy schedule.  Casual response or recipient fatigue after too many text messages. | India | Innovation  Construct:  Innovation Design |
| 6 | Inability to maintain work-personal life boundaries for care providers | Bhat et al., 2018 | CMs experienced some challenges in the use of text messaging, such as maintaining boundaries – both in terms of defining limits to the work day, and setting interpersonal boundaries with their patients. They remarked that text messaging is usually considered a means to communicate with friends and this increases the likelihood of boundary violations when using text messages to communicate with a health care provider. | US | Inner Setting  Construct: Relational Connections |
| 7 | Lack of changing health care worker behavioral attitudes | Onono et al., 2019 | The other thing that makes women not to go the hospital is because the nurses do not show concern. They do not attend to other needs of the women even when they say that they are not feeling well. They just do routine check-ups and tell them to go home. They do not tell you what to do with your problem neither do they refer you like MAccess used to. When you text your problem to MAccess, they refer you to hospital or tell you what to do. These nurses at the hospital just do not care. They just assume your problem and tell you to come back during your next clinic appointment. Moreover, they do not care whether you come back or not...Lack of appetite, child not playing in the womb, too much weight on one side. These are problems that the nurses can solve but they do not care-  . . .When they go to the hospital, they tell them about these problems, but they are not given any solutions. (Apidi, 24 year old with 1 living child). | Rural Kenya | Individuals  Construct:  Motivation (Characteristics Subdomain) |
| 8 | Inclination towards religious beliefs and traditional birth attendants | Onono et al., 2019 | We found that despite the availability of pregnancy and childbirth services, some women were reluctant to use the hospital-based services for several reasons such as religious beliefs and overt preference for the traditional birth attendants (TBAs). Some people are restricted by their religious beliefs, while some have faith with the traditional birth attendants (TBA) and they say that children usually die at the hospital just like they can die at the TBA. | Rural Kenya | Outer Setting  Construct:  Local Attitudes |
| 9 | Unavailability of transport workers | Okonofuna et al., 2023 | Our record indicates that among women requesting transportation through the Text4Life platform, a large proportion (> 90%) were moved to the PHCs with emergency transport. Among those not reached with transport, this was due to late arrival or non-availability of the taxis at the time the request was made. This implies that a system involving the use of multiple and alternative methods of transport and that includes a response from the transport owners regarding their availability at the time of the request would have to be considered. | Rural Nigeria | Outer Setting  Construct: Local Conditions |
| 10 | Low Network Connectivity | Muthelo et al., 2023 | Mother 1 said, “for mom-connect is like am chatting with a person, is like SMS, I just open it and communicate. For pregnancy plus, I must login to their system and access the services, and some of the services I cannot access without data”.  Mother 3 said, “ Though I have used mom-connect during pregnancy, network in this area is a serious challenge”. | Rural South Africa | Inner Setting  Construct: Available Resources (Materials & Equipment) |
|  |  | Kazi et al., 2017 | A higher proportion of those from NAL were more likely to experience network challenges (27.1% vs 14.6%; P<.001). However, 92.1% (35/38) in NAL and 93.3% (14/15) at adjacent site participants were able to overcome the network coverage issues and only 9.2% (26/284) reported of a problem with keeping their phone battery charged overall. | Kenya | Inner Setting  Construct: Available Resources (Materials & Equipment) |
|  |  | Laar et al., 2019 | Our major problem is about the network connectivity. The network is so bad that in some places people have to climb trees or high objects in some communities to call or receive calls… (KIIdHealth workerdJirapa).  Some of us don't have electricity in our communities. Charging phones will be a challenge to us. Anytime the nurses want to call us, and the phone is off they can't reach us (FGDdPregnant women and lactating mothers all districts). | Ghana | Inner Setting  Construct: Available Resources (Materials & Equipment) |
| 11 | Low capacity of phone for multiple apps | Muthelo et al., 2023 | Mother 1: “Pregnancy plus, it needs more space than mom-Connect because when it stops, its needs you to have another app and more space on the phone to accommodate another app”. | Rural South Africa | Inner Setting  Construct: Available Resources (Materials & Equipment) |
| 12 | Lack of consideration of Traditional Healer support on maternal support apps | Muthelo et al., 2023 | Mother 2 reported “oh home, traditional I will start home because we believe in tradition. I have a traditional healer at home, so we can go to the clinic if the traditional healer fails. The traditional healer assesses if they could be able to assist with the maternal and child health problem, and if they ’can’t then, I will go to the clinic”.  CHW 1 “late pregnancy booking is a major challenge because they first consult with the traditional healers and come to the clinic when is already late”. | Rural South Africa | Outer Setting  Construct:  Local Attitudes |
| 13 | Unaffordability of phones and airtime | Laar et al., 2019 | Not all of us have mobile phones…so in this case what are we going to do…and even those who have, some of us can't always buy the airtime (FGDdPregnant women and lactating mothersdall districts).  …so, by introducing technology in maternal and child health, our bigger challenge has to do with the mothers' ability to afford the phones. You want them to go and buy phones, after buying phone you have to put in the credit, so these are some of the challenges I think these mothers are likely to face (KIIdHealth  workerdJirapa).  Some of us don't have proper phones. You know we don't have money to buy phones. It is not only the phones that we do not have, but also money to buy credits to make calls so introducing something like this without the supply of these things will be a problem to some of us (KIIdHealth providerdJirapa). | Ghana | Inner Setting  Construct: Available Resources (Funding) |
|  |  | Zunza et al., 2023 | Many patients explained that they simply could not afford to reg- ularly buy airtime for making calls, often waiting until they had re- ceived their monthly government social security grant to purchase airtime credit.  ‘Yes, it [airtime] is very important and I need it but it's just that I don't have money.’ (Pregnant woman ID: 1240, age 18–24) | South Africa | Inner Setting  Construct: Available Resources (Funding) |
| 14 | Inconsistencies in Explanation of Intervention to Participants | Watterson et al., 2020 | A key barrier was difficulty with consistently offering and explaining interventions to women at intervention clinics. Despite the implementing midwives participating in training at the program’s start and regular visits from the researcher to discuss the program and collect data, evidence suggests that some pregnant women might not have received a clear explanation of the program, or might not have been offered the program even if they registered for ANC at an intervention clinic. One midwife wrote, “[I] sometimes forget to fill in forms but will improve as it becomes part of daily routine.” This quote highlights that implementation of the intervention did not fit into the midwives’ existing workflow, which might have contributed to inconsistent registration, and could explain why the midwives rated registering pregnant women for the program as fairly difficult. | Samoa | Inner Setting  Construct:  Compatibility |
| 15 | Low Awareness of Intervention | Watterson et al., 2020 | In addition, the researcher received responses to some of the text messages asking who had sent the message. This could suggest potential issues such as (1) someone else was using the mobile phone, as phone sharing is a common practice among friends and families in Samoa, or (2) the woman had not understood or had forgotten that she signed up for the messages at the clinic. | Samoa | Inner Setting  Construct:  Access to Knowledge & Intervention |
