# Supplementary material for: Barriers and facilitators of messaging platforms as a means of maternal support and care in rural communities: A systematic review
Source: PLoS One. 2025 Dec 5;20(12):e0336168. doi: 10.1371/journal.pone.0336168 (PMC12680158; doi:10.1371/journal.pone.0336168)
Supplement: S1 Appendix — (DOCX) [file pone.0336168.s005.docx]

**S1 Appendix. Search Strings**

| **Feasibility of implementing messaging platforms as a means of maternal support to enhance antenatal visit rates in rural communities** | | |
| --- | --- | --- |
| Sets 1-3 are the MeSH terms & text words for the population (i.e., maternal support and antenatal care in rural communities). They are combined using OR. | 1."maternal support" or "antenatal care" or "maternal healthcare" or "pregnancy support" or "pregnancy".tw.  **2. [mh “**Maternal Health Services”]  3. #1 OR #2  4. "rural communit*" or "rural population*" or "rural area*" or "remote communit*" or "remote population*" or "remote area*".tw.  5. [mh “Rural Population” or “Rural Health”]  6. #4 OR #5 | **Paricipants (P)** |
| Sets 4-6 are the MeSH terms & text words for the intervention (i.e., messaging platforms). They are combined using OR. | 7."WhatsApp" OR "messaging platform" OR "text messag*" OR "social media" OR “digital platform” OR “instant messag*” .tw.  8. [mh "Telemedicine"]  9. [mh “Social Media”]  10. #7 OR #8 or #9 | **Intervention (I)** |
| Set 11 is the combination of Population and Intervention | 11.Set 11 is the combination of Population and Intervention | **Combination of**  **P & I** |

| **OVID Medline: Publisher, In-Data-Review, In-Process and PubMed-not-MEDLINE records from NLM <1946 to 2025 January 30> \| Imported January 30, 2025** | | | |
| --- | --- | --- | --- |
| **#** | **Search Statement** | **Results** | **Annotations** |
| **1** | ("maternal support" or "antenatal care" or "maternal healthcare" or "pregnancy support" or "pregnancy").tw. | 490426 |  |
| **2** | Maternal Health Services/ | 17334 |  |
| **3** | 1 or 2 | 503059 |  |
| **4** | ("rural communit*" or "rural population*" or "rural area*" or "remote communit*" or "remote population*" or "remote area*").tw. | 85163 |  |
| **5** | Rural Population/ or Rural Health/ | 94657 |  |
| **6** | 4 or 5 | 147919 |  |
| **7** | ("WhatsApp" or "messaging platform*" or "text messag*" or "social media" or "digital platform" or "instant messag*").tw. | 45406 |  |
| **8** | Telemedicine/ | 43098 |  |
| **9** | social media/ | 18993 |  |
| **10** | 7 or 8 or 9 | 93329 |  |
| **11** | 3 and 6 and 10 | 76 |  |
| **12** | limit 11 to yr="2003 -2025" | 74 |  |

| **OVID Embase: Embase Classic+Embase <1947 to 2025 January 30> \| Imported January 30, 2025** | | | |
| --- | --- | --- | --- |
| **#** | **Search Statement** | **Results** | **Annotations** |
| **1** | ("maternal support" or "antenatal care" or "maternal healthcare" or "pregnancy support" or "pregnancy").tw. | 704310 |  |
| **2** | prenatal care/ or maternal care/ or maternal health service/ | 80878 |  |
| **3** | 1 or 2 | 745531 |  |
| **4** | ("rural communit*" or "rural population*" or "rural area*" or "remote communit*" or "remote population*" or "remote area*").tw. | 10660 |  |
| **5** | rural health care/ or rural health/ | 17570 |  |
| **6** | rural population/ | 63967 |  |
| 7 | 4 or 5 or 6 | 152779 |  |
| **8** | ("WhatsApp" or "messaging platform*" or "text messag*" or "social media" or "digital platform" or "instant messag*").tw. | 56933 |  |
| **9** | telemedicine/ | 50997 |  |
| **10** | social media/ | 60976 |  |
| **11** | 8 or 9 or 10 | 130144 |  |
| **12** | 3 and 7 and 11 | 119 |  |
| **13** | limit 12 to yr="2003 -2025" | 119 |  |

| **Scopus: 2003 to 2025 > \| Imported January 30, 2025** | | | |
| --- | --- | --- | --- |
| **#** | **Search Statement** | **Results** | **Annotations** |
| **1** | "maternal support" OR "antenatal care" OR "maternal healthcare" OR "pregnancy support" OR "pregnancy" OR "prenatal care" OR "maternal care" OR "maternal health service" |  |  |
| **2** | "rural communit*" OR "rural population*" OR "rural area*" OR "remote communit*" OR "remote population*" OR "remote area*" OR "rural health care" OR "rural health" OR "rural population" |  |  |
| **3** | "WhatsApp" OR "messaging platform*" OR "text messag*" OR "social media" OR "digital platform" OR "instant messag*" OR "telemedicine" OR "social media" |  |  |
| **4** | 1 AND 2 AND 3 | 334 |  |

| **CINAHL \| Imported January 30, 2025** | | | |
| --- | --- | --- | --- |
| **#** | **Search Statement** | **Results** | **Limiters and Expanders** |
| **S1** | "maternal support" or "antenatal care" or "maternal healthcare" or "pregnancy support" or "pregnancy" | 275,593 | **Expanders** - Apply equivalent subjects  **Search modes** - Boolean/Phrase |
| **S2** | (MH "Maternal-Child Care") OR (MH "Maternal-Child Health") OR (MH "Maternal Health Services") OR "maternal health or maternal care" | 18,003 | **Expanders** - Apply equivalent subjects  **Search modes** - Boolean/Phrase |
| **S3** | (MH "Prenatal Care") OR "prenatal care" | 23,713 | **Expanders** - Apply equivalent subjects  **Search modes** - Boolean/Phrase |
| **S4** | S1 OR S2 OR S3 | 287,312 | **Expanders** - Apply equivalent subjects  **Search modes** - Boolean/Phrase |
| **S5** | "rural communit*" or "rural population*" or "rural area*" or "remote communit*" or "remote population*" or "remote area*" | 56,468 | **Expanders** - Apply equivalent subjects  **Search modes** - Boolean/Phrase |
| **S6** | (MH "Rural Health") OR "rural health" OR (MH "Rural Health Services") | 16,919 | **Expanders** - Apply equivalent subjects  **Search modes** - Boolean/Phrase |
| S7 | (MH "Rural Population") OR "rural population" | 14,533 | **Expanders** - Apply equivalent subjects  **Search modes** - Boolean/Phrase |
| **S8** | S5 OR S6 OR S7 | 66,417 | **Expanders** - Apply equivalent subjects  **Search modes** - Boolean/Phrase |
| **S9** | “WhatsApp" or "messaging platform*" or "text messag*" or "social media" or "digital platform" or "instant messag*" | 45,482 | **Expanders** - Apply equivalent subjects  **Search modes** - Boolean/Phrase |
| **S10** | (MH "Telemedicine") OR "telemedicine" OR (MH "Telehealth") | 36,602 | **Expanders** - Apply equivalent subjects  **Search modes** - Boolean/Phrase |
| **S11** | (MH "Social Media") OR "social media" | 38,231 | **Expanders** - Apply equivalent subjects  **Search modes** - Boolean/Phrase |
| **S12** | S9 OR S10 OR S11 | 80,558 | **Expanders** - Apply equivalent subjects  **Search modes** - Boolean/Phrase |
| **S13** | S4 AND S8 AND S12 | 131 | **Limiters** - Published Date: 20030101-20250129  **Expanders** - Apply equivalent subjects  **Search modes** - Boolean/Phrase |
